# A Comparative Study of the Removal of Smear Layer by Two Endodontic Irrigants and Nd:YAG Laser: A Scanning Electron Microscopic Study

**DOI:** 10.5402/2012/620951

**Published:** 2012-07-16

**Authors:** Seyed Mohsen Hasheminia, Reza Birang, Mahboobe Feizianfard, Mina Nasouri

**Affiliations:** ^1^Department of Endodontics and Torabinejad Dental Research Center, School of Dentistry, Isfahan University of Medical Sciences, Isfahan 817463461, Iran; ^2^Department of Periodontology and Torabinejad Dental Research Center, School of Dentistry, Isfahan University of Medical Sciences, Isfahan 817463461, Iran; ^3^Department of Endodontics, School of Dentistry, Isfahan University of Medical Sciences, Isfahan 817463461, Iran; ^4^School of Dentistry, Isfahan University of Medical Sciences, Isfahan 817463461, Iran

## Abstract

The aim of this study was to compare the effect of 17% EDTA, 5% maleic acid, and Nd:YAG laser on smear layer removal by SEM. Eighty single-rooted teeth were divided into three groups of 25 according to the final procedure for smear layer removal: irrigation by 17% EDTA or 5% maleic acid or Nd:YAG laser irradiation. The other five teeth was used as control. Roots were sectioned into buccal and lingual parts, and smear layer presence was recorded in the coronal, middle, and apical thirds under SEM. Data were analyzed by Kruskal-Wallis, Mann-Whitney, Friedman, and Wilcoxon tests. There was no significant difference between smear layer removal of 17% EDTA and 5% maleic acid. Nd:YAG laser showed the least effect significantly. The coronal part of samples was significantly cleaner than the middle, and the middle was cleaner than the apical section. 17% EDTA and 5% maleic acid were more effective in smear layer removal compared to Nd:YAG laser.

## 1. Introduction

The success of root canal treatment depends on cleaning and disinfection of the canal to perform adequate obturation. The smear layer is an amorphous irregular layer containing inorganic debris as well as organic materials like pulp tissue, odontoblastic process, necrotic debris, microorganisms, and their metabolic products. It appears only on instrumented root canal dentine [1, 2].

McComb and Smith were the initial investigators who found the smear layer on the instrumented root canal walls. They found it irregular, amorphous, and granular when viewed under the SEM [3]. Some investigators believed that the presence of the smear layer helps increase the success rate of endodontic treatment because this layer blocks dentinal tubules and prevents bacterial exchange by reducing dentine permeability. They reported that the presence of the smear layer could stop bacterial migration from dentinal tubules or bacterial invasion into the dentinal tubules [4, 5].

Instead, others focused on the smear layer removal. Brännström and Pérez-Heredia et al. believed that the smear layer feeds microorganisms and helps them colonize [6, 7]. Some researchers have reported that the smear layer prevents or delays action of canal irrigation solutions for disinfection of the bacteria and microorganisms in dentine [8, 9]. Other investigators showed root canal sealers to have a better adhesion to the root canal wall after smear layer removal [10–12].

Different methods have been used to remove the smear layer. Sodium hypochlorite (NaOCl) is a common irrigation solution used in endodontic therapy because it has bactericidal properties and the ability to dissolve organic tissues [13–16], but this solution has no ability to remove smear layer alone. Erickson and Van Meerbeek et al. have reported that maleic acid has an ability to remove the smear layer. Maleic acid is a mild organic acid used as an acid conditioner in adhesive dentistry due to etching action [17, 18]. The most common chelating solution is EDTA, which reacts with calcium ions in dentine and produces soluble calcium chelates [19]. Nygaardostby was the first investigator who used EDTA to clean and shape the canals [20]. Some researchers have reported that alternating the use of EDTA and NaOCl can remove the smear layer in an ideal way [16, 21–23]. Weichman and Johnson were the first researchers who used laser in endodontic treatments [24]. Takeda et al. and Ayad believed that laser could vaporize canal soft tissue and remove smear layer [25, 26]. Levy and Goodis et al. have reported that usage of Nd:YAG laser, when followed by manual filing, can clean root canal walls and remove the smear layer and the other soft tissue from the root canal. They used an Nd:YAG laser to irradiate the dentine of the root canal wall and showed disruption of the smear layer to actual melting and recrystallization of the dentine [27, 28].

The purpose of this study was to compare the in vitro effect of 17% EDTA, 5% maleic acid, and Nd:YAG laser irradiation on smear layer removal of prepared root canal walls by scanning electron microscopy.

## 2. Materials and Methods

In this experimental in vitro study, 80 single-rooted human teeth were selected. The teeth had been recently extracted from patients between the ages of 30 and 40 years because of periodontal diseases and prosthetic reasons. The teeth were radiographed to verify any abnormalities. After cleaning, the teeth were immersed in isotonic saline solution. Then, the crowns of the teeth were dissected by nonstop device (Krupp Dental Dentarapid, Hilzingen, Germany) and diamond disc (D&Z, Darmstadt, Germany). The remaining roots lengths were almost 13 mm.

A no. 15 k-file (Mani, Touchi, Japan) was passed 0.5 mm beyond the apical foramen to ensure patency, and, then, 1 mm was subtracted from the measurement to be used as the working length. Next, apical closure wax was used to obtain the conditions close to the clinical situation. The teeth were instrumented with hand instruments by step-back technique up to no. 40 k-file (Mani, Touchi, Japan) in apical part and up to no. 80 k-file (Mani, Touchi, Japan) in coronal part. Each instrument was used for not more than 10 preparations and then was discarded to have a better control on smear layer production. 1 mL of 5.25% NaOCl was used as canal irrigation solution between every two instrumentations. After canal preparation, teeth were stored in distilled water. They were randomly divided into 4 groups according to the final irrigation solutions or laser irradiation.

*Group 1.* Twenty-five teeth were irradiated with Nd:YAG laser (Fotona Fidelis Plus, Ljubljana, Slovenia, Wave length 1.064 nm). The setting parameters were output power 2 w, pulse energy 120 mj/pulse, and pulse frequency 15 Hz with 300 *μ*m optic fiber with hand circular motion from apical foramen to coronal part of canal in a time duration of 40 seconds (4 times, 10 sec each, with 15 sec intervals to prevent temperature rise). After laser irradiation, the canals were irrigated with 5 mL of distilled water and were kept in it. 

*Group 2.* Twenty-five teeth canals were irrigated for 1 min with 5 mL of 17% EDTA (Merck, Germany) buffered up to ph: 7.8. Then the canals were irrigated with 5 mL of 5.25% NaOCl, and finally irrigation was done with 2.5 mL of distilled water to remove any effects of irrigants. 

*Group 3.* Twenty-five teeth were irrigated for 1 min by 5% maleic acid (Merck, Germany), then the canals were irrigated with 5 mL of 5.25% NaOCl, and finally irrigation was done with 2.5 mL of distilled water to remove any effects of irrigants. 

*Group 4 (Control).* Five teeth were washed by 5 mL of 5.25% NaOCl for 1 min as a final irrigation solution and then by 2.5 mL of distilled water.

All irrigations were done by the needle gauge no. 30 to penetrate to the apical third of the canals.

Teeth were longitudinally bisected into buccal and lingual parts by wedging process with the help of spatula after a shallow groove preparation by diamond fissure bur (Teeskavaan, Tehran, Iran). Then, one half of each root was selected for processing as follows.

1. Double fixation with 5% Glutaraldehyde (2-3 hours), Phosphate buffer rinsing (2-3 times), 1% Osmium tetroxide (2-3 hours), Phosphate buffer rinsing (2-3 times).
2. Dehydration by Ethylic alcohol: 30% for 10 minutes, 50% for 20 minutes, 70% for 20 minutes, 90% for 30 minutes, 100% for 30 minutes, 100% for 30 minutes.
3. Drying with liquid CO_2_ for 30 minutes.

After processing, samples were coated with gold palladium by E5200 sputter coater Bio-rade, placed into the SEM device (Cam scan MV 2300, Oxford Instrument, UK) and scanned in three coronal, middle, and apical parts. Finally, photomicrographs were taken in ×2500 magnification.

Data were blind and observed by two independent researchers. They observed the photographs and scored them using an eight-scale score that had been designed by Khademi et al. [29].

**Score 1**

The surface is devoid of debris and smear layer.

**Score 2**

The surface is devoid of smear layer, but little debris is observed.

**Score 3**

The surface has been cleansed, but both smear layer and debris are dispersedly observed.

**Score 4**

The surface has been cleaned, but the level of smear layer and debris is also noticeable.

**Score 5**

The clean surface is a bit greater than unclean surface.

**Score 6**

Almost half of smear layer and debris is removed.

**Score 7**

Greater parts of smear layer and debris are left.

**Score 8**

The surface is completely covered with smear layer and debris.

Finally, the data were analyzed by Kruskal-Wallis, Mann-Whitney, Friedman, and Wilcoxon tests using SPSS software version 11.5.

## 3. Results

**Figure 1:**
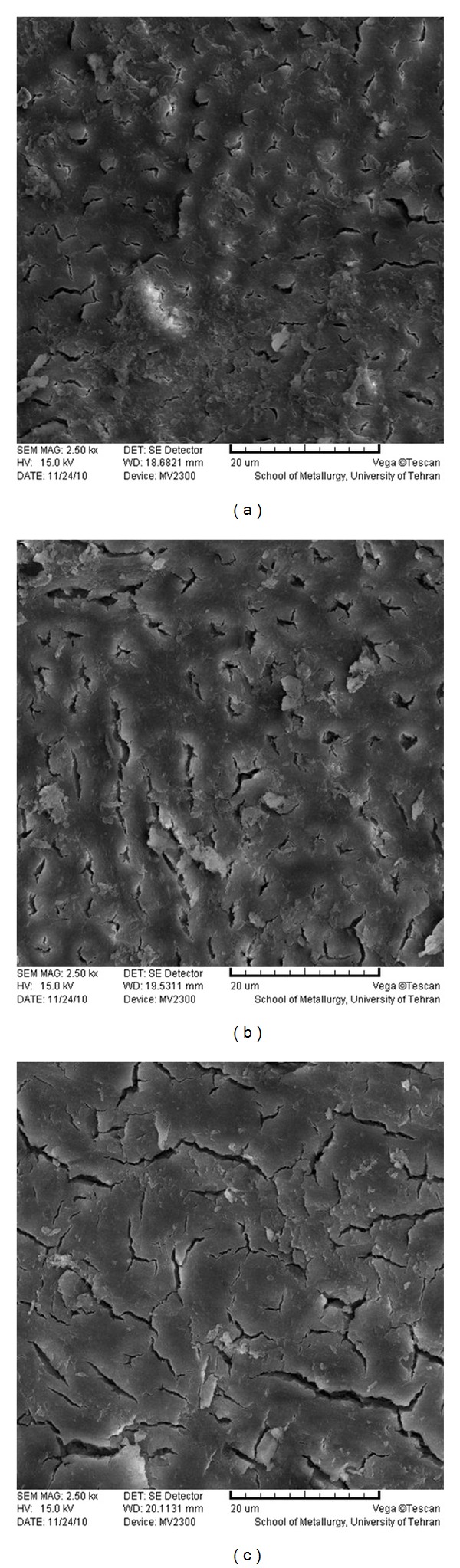
SEM photomicrographs of coronal (a), middle (b), and apical (c) parts of the root canal treated by 5.25% NaOCl (×2500).

**Figure 2:**
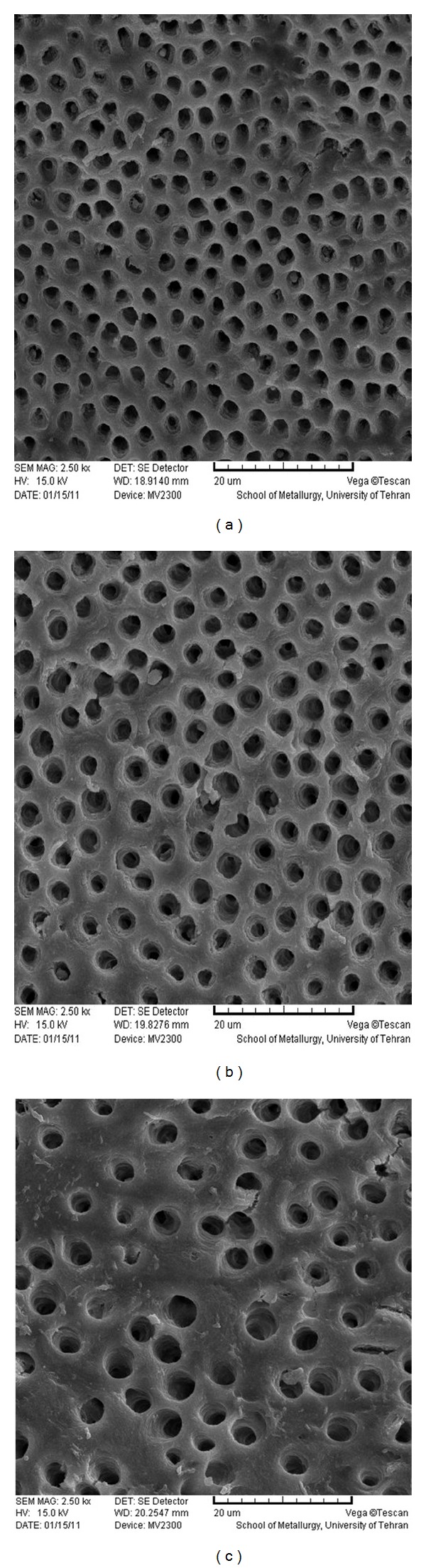
SEM photomicrographs of coronal (a), middle (b), and apical (c) parts of the root canal treated by 17% EDTA (×2500).

**Figure 3:**
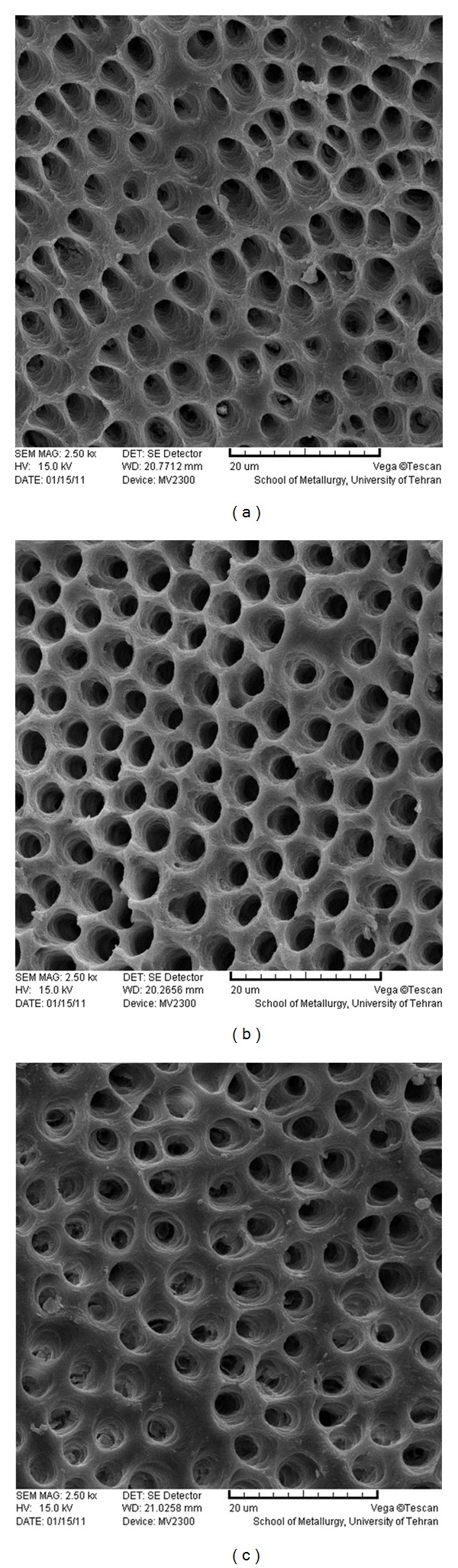
SEM photomicrographs of coronal (a), middle (b), and apical (c) parts of the root canal treated by 5% maleic acid (×2500).

**Figure 4:**
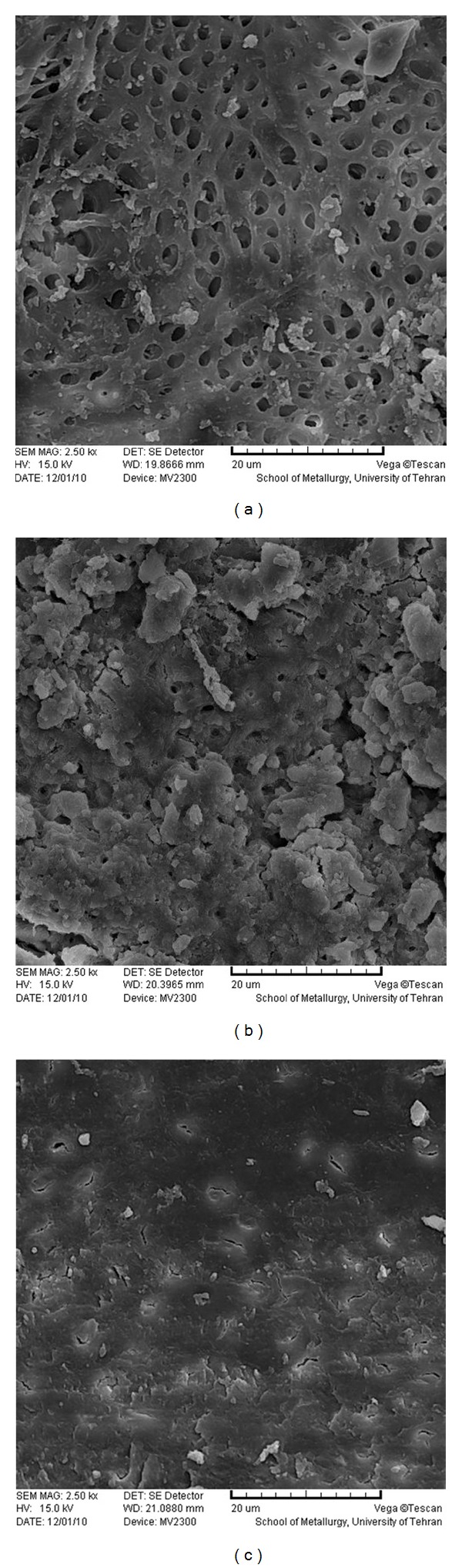
SEM photomicrographs of coronal (a), middle (b), and apical (c) parts of the root canal treated by Nd:YAG laser (×2500).

The mean score and *P* value for the removal of smear layer are presented in Tables 1 and 2 and Figures 1 to 4.

Krusal-Wallis and Mann-Whitney tests showed no significant difference between EDTA/Maleic acid (*P* = 0.409) and Nd:YAG laser/Control (*P* = 0.157) groups in smear layer removal. However, a significant difference was found between EDTA/Nd:YAG laser (*P* < 0.001) and maleic acid/Nd:YAG laser (*P* < 0.001) in smear layer removal. Two Solutions showed more effect in smear layer removal than laser significantly, despite different areas of the canals (coronal, middle, and apical).

Friedman and Wilcoxon tests showed a significant difference between different areas of the canal in each group. Coronal area of Nd:YAG laser samples carried less smear layer than the middle area and it had less smear layer than the apical area (smear layer removal: coronal > middle > apical).

17% EDTA showed equal effect of smear layer removal in coronal and middle areas. These two areas were cleaner than the apical area (smear layer removal: coronal = middle > apical).

Also with 5% maleic acid, the coronal area was cleaner than the middle, and the middle area was cleaner than the apical area from smear layer (smear layer removal: coronal > middle > apical).

Regardless of the technique used in smear layer removal, with the comparison between the three areas of canals, coronal areas in all groups were cleaner than the middle ones and the middle ones carried less smear layer than the apical areas (smear layer removal: coronal > middle > apical).

## 4. Discussion

The findings of the present study suggest that there was no significant difference between EDTA and maleic acid, indicating that both solutions have the same effect on removal of smear layer, but the ability of Nd:YAG laser in removal of smear layer was less than two solutions significantly.

da Silva et al. flashed 14.3% EDTA into root canal, left it for 3 min, and concluded that this irrigation solution is capable of removing smear layer from root canal wall [30]. Sen et al. demonstrated that the removal of smear layer by different concentrations of EDTA (1%, 5%, 10%, and 15%) is not significantly different [31]. According to Wadhwani et al. there was no significant difference between 17% EDTA solution and 19% EDTA gel in smear layer removal [32]. Calt and Serper's investigation on 1 and 10 min application time has shown that the ability of 17% EDTA in 1 min application time is agreeable and prevents harmful consequences such as excessive erosion, enlargement of dentinal tubule openings, and deterioration of the dentinal surface [33].

It has been suggested in some previous studies that maleic acid is effective in smear layer removal, and 5% concentration of this solution was recommended [34], which confirms the results of this study. Comparison of maleic acid and EDTA in the Prabhu et al. and Ballal et al. studies showed more effect of maleic acid on the middle third and apical third segments of the root canal, something inconsistent with the present study probably because of different score rating and needle gauge used in the mentioned study. The other studies showed that maleic acid has a less toxic effect on tissue than EDTA. So, they suggested that maleic acid can be a fine replacement for EDTA [34–36].

Since laser has shown the different applications in dentistry, the researchers have started to use its power in smear layer removal. A thin fiber development for the Nd:YAG laser stimulated reporters to study it more. Different findings were achieved by different researchers.

Goya et al. reported that Nd:YAG laser irradiation on root canal dentine leads to eliminate smear layer from root canal wall, something in controversy with the results of the present study due to their applying 14% EDTA (as a final irrigation solution) before laser irradiation. However, it possibly may be due to interference with laser effects [37].

Takeda et al. focused on the successful implementation of Nd:YAG laser. The present study suggested 20% smear layer removal. There are differences in the results of Takeda et al. and the present study, and it is because of different laser parameters and score ratings applied in these studies [38].

In a study of Nd:YAG laser, 15% EDTA and a few solutions were examined by Gurbuz et al. and it was shown that the Nd:YAG laser and EDTA were the most effective ways for removal of smear layer. They also found no significant difference between them [39]. It does not establish the results of the present study based on the following reasons: the difference between laser parameters, the difference between score rating, and SEM evaluation in only one area in Gurbuz et al.'s study instead of three areas in the present study.

Some researchers reported that using Nd:YAG laser is not an effective way to remove the smear layer, consistent with the results of the present study [40–42].

The findings of the present study showed that Nd:YAG laser has less ability in smear layer removal in comparison with EDTA and maleic acid. It should be noted that in some SEM photographs of laser specimens, smear layer had been removed and melted. So, it can be clearly demonstrated that the laser capability for smear layer removal strongly depends on the parameters and the laser exposure technique.

Finally, the comparison between laser and acidic solutions for smear layer removal may not be exactly a right thing to do since the solutions have an ability to solve smear layer but laser melts, vaporizes, and recrystallizes smear layer. Meanwhile, the scores commonly used in these kind of studies have been mainly designed for smear layer removal and not its melting.

At the end, the main aim of the removal of smear layer is to eliminate microorganisms from root canal and to disinfect open dentinal tubules. So, if laser exposure can reduce the number of microorganisms and their products and partly open dentinal tubule orifices, it may yield the same results of smear layer removal by acidic solutions.

Comparing the different regions of the root canal walls, the results of this study showed that smear layer removal of the coronal and the middle segments in 17% EDTA was equal and these two areas were cleaner than the apical third of the root canal. 5% maleic acid and Nd:YAG laser samples similarly showed cleaner coronal surface than the middle and the apical thirds, respectively.

Numerous investigations confirmed that the coronal and middle areas of the canals irrigated by EDTA were cleaner than the apical part [25, 31, 34–36, 43], something consistent with the finding of the present study.

The findings of Nd:YAG laser samples in the present study were consistent with those of Zhang et al. and Barbakow et al. On the contrary, the findings in the present study were not consistent with the study of Kivanç et al. [40–42].

This study concluded that the coronal third and middle third were cleaner than the apical third, and this may be due to the lack of solution penetration or incorrect laser exposure in this area (apical third). It is clear that due to the wide openings of dentinal tubules in coronal and middle thirds, laser and solutions can act more effectively.

## 5. Conclusions

As a result of this study, we can conclude that 17% EDTA and 5% maleic acid were more effective than Nd:YAG laser in removal of smear layer from root canal walls.

## Figures and Tables

**Table 1:** Analytical statistics in different groups and their areas.

Groups	Areas	Mean score	Standard deviation	Mean score (total)	*P* value
Nd:YAG laser	Coronal	6.10	2.37	7.05	0.00
	Middle	7.30	1.06		
	Apical	7.76	0.45		

17% EDTA	Coronal	1.20	0.43	1.33	0.00
	Middle	1.14	0.30		
	Apical	1.66	1.02		

5% Maleic acid	Coronal	1.06	0.21	1.62	0.00
	Middle	1.14	0.30		
	Apical	2.66	2.01		

Control	Coronal	8.00	0.00	7.86	0.05
	Middle	8.00	0.00		
	Apical	7.60	0.41		

**Table 2:** *P* values obtained with the comparison between the groups at the apical, middle, and coronal areas.

Groups	Overall	Coronal	Middle	Apical
Laser Nd:YAG-EDTA	<0.001	<0.001	<0.001	<0.001
Laser Nd:YAG-Maleic acid	<0.001	<0.001	<0.001	<0.001
EDTA-Maleic acid	0.40	0.07	1.00	0.22
Maleic acid-Control	<0.001	<0.001	<0.001	<0.001
EDTA-Control	<0.001	<0.001	<0.001	<0.001
Nd:YAG laser-Control	0.157	0.049	0.096	0.327
